# Mining the TRAF6/p62 interactome for a selective ubiquitination motif

**DOI:** 10.1186/1753-6561-5-S2-S4

**Published:** 2011-05-28

**Authors:** Trafina S Jadhav, Marie W Wooten, Michael C Wooten

**Affiliations:** 1 Program in Cellular and Molecular Biosciences, Department of Biological Sciences, 331 Funchess Hall, Auburn University, Auburn, AL, 36849, USA; 2 Deceased

## Abstract

A new approach is described here to predict ubiquitinated substrates of the E3 ubiquitin ligase, TRAF6, which takes into account its interaction with the scaffold protein SQSTM1/p62. A novel TRAF6 ubiquitination motif defined as [–(hydrophobic)–k–(hydrophobic)–x–x–(hydrophobic)– (polar)–(hydrophobic)–(polar)–(hydrophobic)] was identified and used to screen the TRAF6/p62 interactome composed of 155 proteins, that were either TRAF6 or p62 interactors, or a negative dataset, composed of 54 proteins with no known association to either TRAF6 or p62. NRIF (K19), TrkA (K485), TrkB (K811), TrkC (K602 and K815), NTRK2 (K828), NTRK3 (K829) and MBP (K169) were found to possess a perfect match for the amino acid consensus motif for TRAF6/p62 ubiquitination. Subsequent analyses revealed that this motif was biased to the C-terminal regions of the protein (nearly 50% the sites), and had preference for loops (~50%) and helices (~37%) over beta-strands (15% or less). In addition, the motif was observed to be in regions that were highly solvent accessible (nearly 90%). Our findings suggest that specific Lysines may be selected for ubiquitination based upon an embedded code defined by a specific amino acid motif with structural determinants. Collectively, our results reveal an unappreciated role for the scaffold protein in targeting ubiquitination. The findings described herein could be used to aid in identification of other E3/scaffold ubiquitination sites.

## Background

The process of signal transduction is dependent upon specific protein-protein interactions, with a small number of proteins – called 'hubs' possessing the ability to interact with many different partners to form multimeric signaling complexes referred to as signalsomes. These hubs mediate interactions by their modular protein domains that confer specific binding activity to their interacting partners. Protein p62 contains several structural motifs that allow it to function as a hub for protein-protein interactions. These motifs include an acidic interaction domain (AID/ORCA/PC/PB1) that binds the aPKC, a ZZ finger, a binding site for the RING finger protein TRAF6, two PEST sequences, and the UBA domain [1]. In this study, we focused on the mechanism by which TRAF6, along with p62, targets specific Lysines for ubiquitination. The addition of ubiquitin, a 76 amino acid protein, occurs via a three step process involving the concerted action of the E1 ubiquitin activating enzyme, E2 the ubiquitin conjugating enzyme and E3 the ubiquitin ligase. Substrates are recognized by different E3s or E2/E3 complexes. The incorporation of a scaffold into the model, such as p62, would serve as a crucial bridge between enzyme (E3 ligase, TRAF6) and its substrate(s) and provide specificity for enzyme-substrate reactions [2]. Thus substrate recognition, site selection and ultimately the ubiquitination reaction result from the activation of the E3 once docked on the scaffold. In support of this model, inhibition of TRAF6/p62 interaction blocks the activity of TRAF6 along with diminished K63-ubiquitination of its target substrate [3].

**Figure 1:**
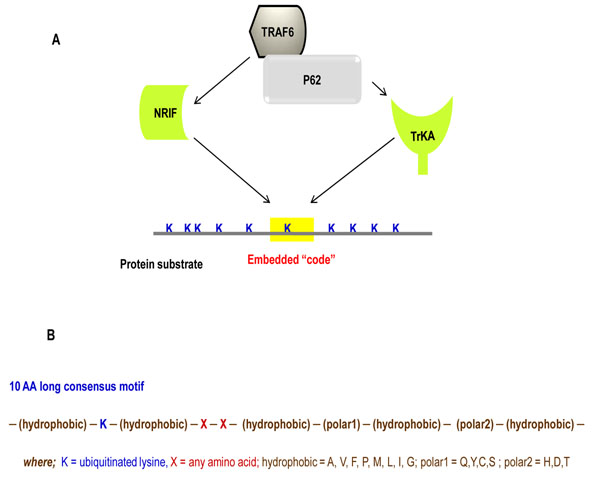
**Model illustrating the interaction between the ligase and scaffold in directing substrate ubiquitination. A:** Schematic representation of the means by which the E3 ligase, TRAF6, interacts with the scaffold, p62, and selects a specific Lysine for ubiquitination. **B:** The sequence of the consensus motif identified in TRAF6/p62 substrates.

Two TRAF6/p62 substrates, tyrosine receptor kinase A (TrkA) [3] and neurotrophin receptor interacting factor (NRIF) [4] (Figure 1A) were identified by us. Mutagenesis studies verified that both of these proteins are K63- polyubiquitinated at specific Lysine residues, K485 in TrkA and K19 in NRIF. The RING finger domain of TRAF6 ligase is known to be responsible for its catalytic E3 ligase activity [5] and also is responsible for binding of the substrate [6], which then mediates polyubiquitination of target proteins. The modular scaffold protein p62 provides the platform for the transfer reaction to occur [3]. Other studies have demonstrated TRAF6-mediated polyubiquitination including TRAF6 auto-ubiquitination, NEMO [5], TAB2 and TAB3 [7]. These reactions, however, have not been shown to require p62 to mediate the modification. Moreover, like TRAF6, there are many reported E3 Ub ligases whose potential pool of biological targets is unknown.

There are approximately one thousand reported E3 ubiquitin ligases in eukaryotes. The preferred substrates for most of these ligases remain largely unknown. Moreover, it remains unclear how among the many Lysines (K) found in an ubiquitinated protein only a few are selected for post-translational modification. Like all signal transduction processes, ubiquitination is a result of specific protein-protein interactions and biochemical reactions occurring at the sub-cellular locales, mediated by modular protein domains. Furthermore, E3 ligases and scaffold proteins interact with numerous binding partners through their multi-domain structures. SQSTM1/p62 is one such scaffold that is known to be a crucial for bridging the E3 ligase, TRAF6 and its substrates while at the same time providing specificity for the enzyme-substrate reactions. We propose that the scaffold plays an integral component in activation of the E3 and may target the specificity of the ubiquitination reaction [3,4]. Therefore, the scaffold TAB 2 and TAB3 which also interact with TRAF6 [7] would mediate ubiquitination of a different set of substrates, compared to the interaction of TRAF6 with the scaffold p62. These interactions would provide the needed specificity for formation of a signalsome in response to different receptors and external stimuli.

There are approximately 617 genes encoding putative ubiquitin (Ub) E3s compared to the 518 genes reported for protein kinases [8]. Preferred substrates for most of these enzymes remain unknown. The biological importance of E3s requires understanding the site selection process involved in substrate recognition during the ubiquitination reaction. Eukaryotic cells express a single ubiquitin-activating enzyme (E1) that activates free ubiquitin for subsequent transfer to one of approximately 50 ubiquitin-conjugating enzymes (E2) [9]. Ubiquitin E3 ligases recruit both substrate and activated ubiquitin to mediate the transfer of the ubiquitin molecule to the targeted protein either directly or with the help of E2 enzymes [10]. The substrate specificity of the ubiquitination process occurs at the level of the E3 ubiquitin ligases. A large numbers of cellular proteins are known to be ubiquitinated and correspondingly, there are large numbers of E3 ligases with a diverse range of substrates. With fewer than 1% of the cellular proteins being ubiquitinated at a given time on a select number of Lysine residues, our understanding of the ubiquitination process is still in its infancy. A number of *in vivo* and *in vitro* methods have been employed to identify ubiquitinated substrates and their sites, including proteome-scale analyses of the substrates [11-13]. All these methods are time-consuming, labor-intensive, and expensive. In addition, they are focused on characterizing the ‘ubiquitinated proteome’ rather than studying single enzyme substrates. In contrast, computational approaches represent promising alternative methods for identification of ubiquitination sites would be of great value to the field. Until recently, no consensus amino acid motif had been reported for a single ubiquitin ligase. The reported biological specificity seems to be associated with substrate selection. This observation prompted us to hypothesize that there exists an embedded code specified by a string of amino acids that is read by E3 ligase in the target substrate. Employing this hypothesis, we developed a method to predict putative TRAF6/p62 ubiquitination sites. To facilitate identification of the putative consensus motif(s) within a substrate protein, a brute-force search algorithm was designed and implemented to search for the site.

As the starting point, we examined protein sequences of two known TRAF6/p62 substrates, TrkA and NRIF. This initial analysis concentrated on target ubiquitination sites selected to optimize our search for any potential consensus motif. Examination of flanking residues surrounding the target Lysine revealed the presence of a likely consensus motif, which was then used to screen additional protein sequences derived from the Trk receptor family. Ubiquitination sites in TrkB and TrkC proteins were first identified *in silico*[*14*] and then confirmed through site-directed mutagenesis and functional testing. The final analysis identified a 10-amino acid long sequence of [-hydrophobic – k – hydrophobic – x – x – hydrophobic - polar1 – hydrophobic - polar2 – hydrophobic -]. The hydrophobic amino acids included Alanine, Leucine, Valine, Methionine, Glycine, Phenylalanine, or Isoleucine. The polar1 amino acids included Glutamine, Tyrosine, Cysteine, or Serine and polar2 included Histidine, Aspartic Acid, or Threonine (Figure 1B).

## Methods

The overall goal of this study was to test the hypothesis that TRAF6 and p62- interacting proteins are putative E3 ubiquitin ligase substrates sharing a common target ubiquitination motif. Because our putative motif was pattern based and did not exhibit a fixed amino-acid sequence, we organized our project in a step-wise fashion. First we asked if the putative motif was broadly present within TRAF6 and p62-interacting proteins. This search included questions regarding the form and complexity of extant motifs, as well as, what subset of amino acids served as the core components. Next we attempted to evaluate the uniqueness of the motif forms identified. This was accomplished by both screening for the occurrence of the motif pattern among unrelated proteins and by estimating the probability of observing such motifs within a randomly generated amino acid sequence. In the final step, we further characterized our target motifs relative to specific characteristics known to be associated with protein function.

### Database preparation

To permit testing of our motif pattern, a database containing 209 proteins was organized. Amino acid sequences were obtained from the National Center for Biotechnology Information (NCBI) protein repository. The proteins sequences were from a mixture of mammalian sources with human-derived proteins preferentially used when available. A full listing of proteins is provided in Jadhav [15]. Proteins within the full database were subdivided into two groups, an experimental dataset containing known or likely TRAF6 / p62 interactors (n = 155) and a negative dataset containing randomly selected proteins with no known association (n = 54). The experimental dataset was further divided into five groups depending on the protein’s likelihood of being a TRAF6/p62 substrate. The subsets ranged from known ubiquitinated substrates with mapped sites to either TRAF6 or p62 interacting proteins. All known TRAF6/p62 substrates with verified ubiquitination (Ub) sites were placed in group I. Group II contained known and tested substrates of the TRAF6 E3 ligase whose target Lysine Ub site(s) were not mapped nor identified and their interaction status with p62 was unknown. TRAF6- or p62- interactors identified from various protein-protein interaction databases [HPRD [16], and BioGRID [17] and EntrezGene [18] formed Groups III and IV, respectively. Finally, Group V was comprised of proteins from the insoluble Formic acid (FA) fraction of the brain from p62 knockout mice. These proteins were included because the trafficking and turnover of these proteins are p62 dependent, and therefore, these proteins may also be either TRAF6 or p62 substrates. The negative dataset contained 54 proteins with no known evidence of interaction with either TRAF6 or p62 proteins. This dataset was used both for control comparisons and as a test group for the search algorithm.

### Motif search protocol

Amino acid sequences of the 209 database proteins were searched for matches to all possible combinations of the putative motif using a brute-force approach. An exhaustive search approach was chosen over existing algorithms for two reasons: most existing motif programs are either limited to searching for individual motif sequences which is inconsistent with our variable pattern or programs that do include variation (e.g. SLiMSearch) are designed to only report perfect matches. Because we were interested in evaluating the entire distribution of matches, particularly those found to be near-perfect (7, 8 or 9 positions), we felt that a brute force approach which identified all potential matches would be most informative. To conduct this search, a file containing all unique combinations of seven variable positions in the 10 amino acid long target motif (hydrophobic – k – hydrophobic – x – x – hydrophobic – polar1 – hydrophobic – polar2 – hydrophobic) was generated (Figure 1B). Ideally our search would have included all possible combinations of amino acids hypothesized to occur in each of the 10 motif positions. Such an approach would, however, have required examining over 80 million character strings at each lysine residue; certainly possible but not practical. As a compromise, we chose to search using only the seven well defined positions and to conduct an *a posterior* analysis of the two positions (x) that could contain any amino acid. The results from this analysis yielded no consistent amino acid pattern for these two sites (data not shown), suggesting that our decision was appropriate. Excluding the two x positions and the fixed Lysine, a total of 201,684 unique seven position motifs were possible. Two computer-based search algorithms were employed to facilitate screening for the presence of motifs. The first program, MotifMaker, was a simple looping program that generated and stored all 201,684 potential motifs combinations. The second program, MotifFinder, implemented a brute-force search algorithm for all possible motif constructs. The analysis started by identifying and counting each ***K***(Lysine) within the target peptide. Any ***K***within 8 residues from the carboxyl end was excluded because it would be impossible for it to be a full motif. The motif search then proceeded by temporarily storing the ***K-1*, *K*+*1*, *K*+*4…K*+*8***amino acids for each ***K***as a character string and comparing this string to each of the 201,684 potential motif patterns. A step-up procedure was used to determine the best fit among the potential motifs. For each ***K***, a counter would be initially set at “zero” matches. The counter would be progressively updated as positive matches between the target string and potential motifs were encountered. The matching motif would then be stored in the computer memory. By searching all possible motif combinations, this approach ensured that the maximum ‘best match’ motif was identified. In motifs that matched at all 7 variable positions, a perfect match was identified. In motifs with less than perfect matches (6, 5, 4,…1), the algorithm ensured that no motif with a greater number of matching locations could be found. The procedure was repeated at each ***K***within the target peptide until all positions had been searched. Information on protein ID, the location of each ***K***, the “best match” motif pattern for the amino acids surrounding each ***K*,** the corresponding motif pattern and the total count of positive hits was stored as output for each protein. Both programs were developed and executed using MATLAB® V6.5 (MathWorks Inc., Natick MA) and are freely available from the corresponding author (MCW).

To verify the accuracy of our search results both the experimental and negative datasets were submitted to SLiMSearch [http://www.southampton.ac.uk/~re1u06/software/slimsearch/index.html]. This program searches pre-defined SLiMs (Short Linear Motifs) in a protein sequence database and allows individual residues to vary as defined by the user. We utilized this program to search for perfect (10 amino acid) matches which we then compared to the results provided by our MotifFinder algorithm.

### Statistical analysis

Results from the MotifFinder search of the 209 study proteins were compiled as frequency distributions representing the number of positive motif “hits” ranked from 0 (no matches) to 7 (perfect match). Standard descriptive statistics (mean, mode, variance, skewness and kurtosis) were generated for each distribution. Of particular interest were the estimates of kurtosis and skewness [19] which were derived for each full empirical distribution and for distribution subsets (5, 6, 7 hits). Statistical comparisons between the experimental and negative datasets were made using Chi-squared analyses (with Yates’ correction and one degree of freedom) or t-tests [20]. Statistical calculations were generated using SAS 9.1 (SAS Institute Inc., Cary NC), Minitab 16 (Minitab Inc., State College, PA) or by hand.

A randomization approach was used to further evaluate the uniqueness of the consensus motif and to provide a basis for initial estimates for the probability of pattern occurrence. This procedure involved the creation and searching of 999 amino acid sequences which were randomly generated using a parameter space designed to reflect the information content found in the original experimental dataset. The procedure was initiated by identifying the frequency of occurrence for each amino acid contained in the 155 proteins of the experimental set. This empirical distribution was used as the source for random draws of amino acids (with replacement) that were used to construct 999 sequences. Each of these sequences was 565 amino acids in length which corresponded to the median size of actual proteins in the experimental dataset. The entire randomized dataset was searched for motif matches using MotifFinder and the results compiled / analyzed as described above.

### Sequence logos

To aid in visual evaluation of putative motifs, sequence logos [WebLogo; 21, 22] were created for the sets of 7 (perfect fit) and 6 (near fit) amino acid motifs identified. This program generates a display of the amino acid distribution surrounding the core motif Lysine. The height of each letter in a stack is proportional to its frequency at that position in the motif set. Letters were further sorted with the most frequent amino acid on top.

### Secondary structure prediction

PSIPRED [23,24] was used to predict secondary structures. PSIPRED uses neural networking and searches for homologous proteins with known structures to determine the most likely structure at each residue position. Predictions of disorder regions at predicted ubiquitinated sites were made using the Metaserver of Disorder (MeDor) [25]. MeDor collects disorder and secondary structure predictions from servers available on the web and generates a graphical output. The web-based database SMART [26] was used to predict signaling domains within the protein sequences identified as containing strong motif patterns. The SABLE server was used to predict from sequence secondary structures and solvent accessibilities, with the goal of identifying potential characteristics of predicted Ub sites in terms of structural profiles [27].

## Results

### Results from motif search and statistical analyses of hit distributions

The 155 proteins in the experimental dataset ranged in length from 103 to 4,572 amino acids with a median length of 565 amino acids (mean = 755.9 ± 609.5). A total of 7,592 Lysine residues (range = 1 – 260 per sequence) were found within the 155 sequences. This produced a median count of 33 Lysines per protein with one Lysine residues occurring on average every 19.7 residues (median = 17). The 54 protein negative dataset contained fewer total Lysines (2,220) but median values were similar between the two datasets. The negative dataset proteins ranged from 147 to 2,115 amino acids in length (median = 584; mean = 738.0 ± 496.7). Lysines ranged from 4 to 151 per protein with a median count of 29 and an average of one Lysine per 18.7 residues. Of the 7,592 total Lysines in the experimental dataset, 7,494 (98.7%) were located at positions within the sequence that could allow for a full 10 amino acid motif. In the negative dataset, 2,209 of 2,220 total Lysines (99.5%) were similarly available.

**Figure 2:**
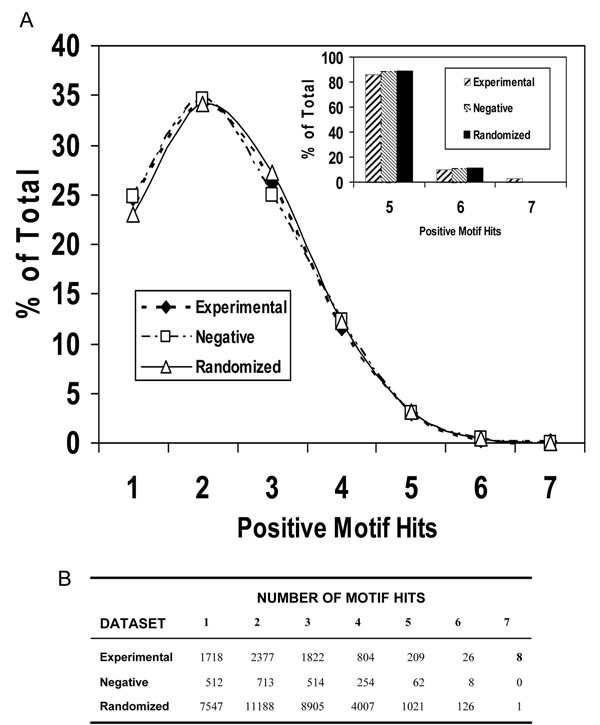
**Motif match search results. A:** Percentage distributions for positive matches at 1, 2, 3, 4, 5, 6, and 7 of the variable sites within the 10 amino acid long putative motif. The number of proteins in each dataset was: Experimental = 155, Negative = 54, Randomized = 999. **(insert).** Percentages for a subset of positive hits (5, 6, 7) hypothesized to be biological meaningful. **B:** Actual counts of the number of matches observed in each dataset for each level of positive match.

**Figure 3:**
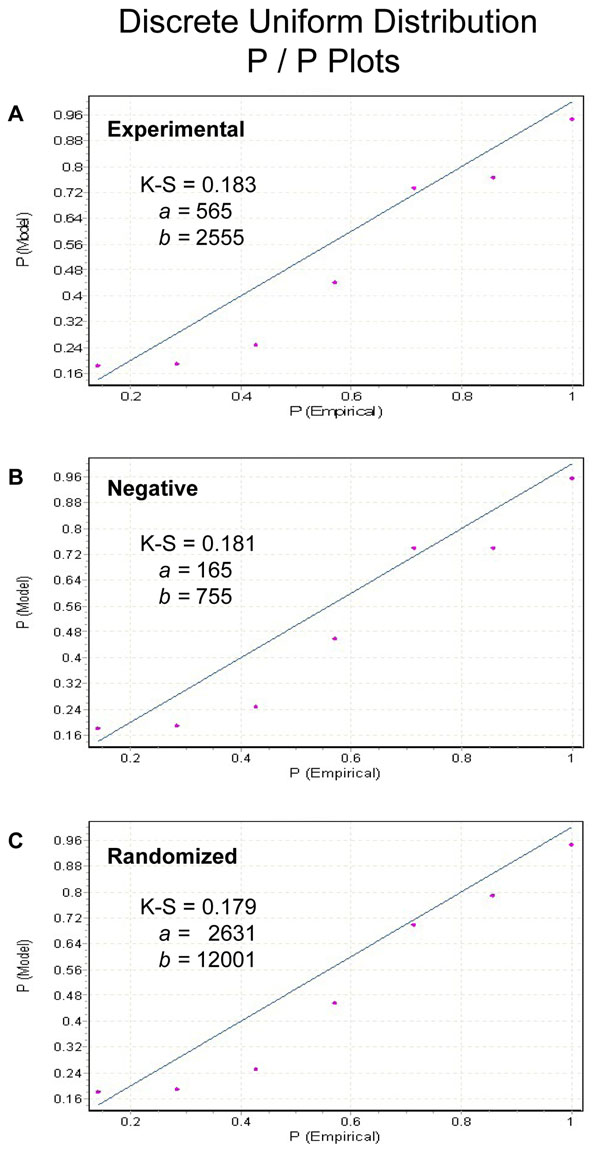
**Probability – Probability (P/P) plots illustrating the relationships between values predicted under Discrete Uniform Distribution models and empirical motif match results. A:** P/P plot for the experimental dataset match distribution. **B:** P/P plot for the negative dataset match distribution. **C:** P/P plot for the randomized dataset match distribution

As expected given the large number of motif combinations analyzed (201,684 variants), results from the motif searches yielded a wide distribution of matches in the regions surrounding individual Lysines (Figure 2). The number of positive matches ranged from 1 to 7 in the experimental proteins and 1 to 6 in the negative dataset. A score of 7 “hits” represented a perfect match in that an appropriate amino acid was found at each of the variable residue locations surrounding the core Lysine. The 7 position matches found using MotifFinder were identical to those produced using SLiMSearch, thus verifying the accuracy of our programming. Both visual inspection and statistical analysis indicated, not surprisingly, a strong similarity between the overall distributions of motif matches for the experimental and negative datasets (Figure 2A). Based on Kolmogorov-Smirnov statistics the best-fitting model for both distributions, as well as the randomized dataset, was the discrete uniform distribution. Figure 3 illustrates the fit of each distribution to its predicted values. The overall similarity among the dataset match distributions was driven by the domination of low accuracy hit (1, 2, 3 or 4) frequencies in both distributions (Figure 2B: 96.5% of experimental dataset matches; 96.6% of negative). This condition was reflected in skewness and kurtosis estimates for each distribution. Both datasets were positively skewed (estimate = 0.31 for both). Consistent with their fit to a discrete uniform model, both distributions were platykurtotic (-1.96 for experimental; -1.81 for negative). Chi-squared goodness-of-fit tests were used to examine how well the observed data from the negative and experimental datasets agreed. Using multiple analyses we investigated whether the distribution of positive hits in the negative dataset conformed to the positive hits in the experimental dataset. These analyses included comparisons of the full sets of positive matches (1-7) and smaller subsets (4, 5, 6, 7 or 5, 6, 7). Because some expected cell counts were below 5, probabilities were estimated using resampling techniques. No comparisons yielded Chi-squared estimates considered to be indicative of departure from expectations (*P* >*0.3* for all tests).

Because low accuracy hits dominated all statistical comparisons and potentially masked meaningful differences, we next evaluated a subset of the distributions corresponding to match values > 4 (Figure 2B and insert). Our assumption was that matches to the putative motif at 10, 9 or 8 positions might be biologically meaningful while those below these values were likely spurious. Proportions of 5 match hits were consistent between the two datasets (experimental = 86.0; negative = 88.6), however, closer examination of the 6 and 7 hit regions of the distributions revealed a strong dichotomy. Perfect matches to all 7 variable residues of the putative motif (Figure 2B) were only found among the experimental proteins. One protein, TrkC, contained two perfect match sequences. No perfect matches were identified within the negative dataset. Twenty-six matches at 6 variable sites were reported from the experimental dataset and 8 from the negative. Because differences between the dataset results were primarily at the 6 (near perfect) and 7 (perfect) matches and little consistency could be identified in the sequence patterns among the level 5 motif matches, all further analyses concentrated on 6 or 7 match results only.

While informative, direct comparisons between the experimental and negative data sets did suffer from limitations. There existed an obvious bias within the experimental dataset because it was purposely assembled with proteins known or suspected to contain some form of our putative motif. This is not an unusual situation as many motif discovery studies use as input, sets of sequences hypothesized to contain a biologically important sequence pattern. These sequences are then searched for patterns that are unlikely to occur by chance. We attempted to estimate the probability of encountering perfect or near perfect matches within our datasets by chance using a randomized data approach. For this the frequency of amino acids found in the experimental dataset was used to randomly generate 999 test “proteins”. These 999 artificial proteins were then screened using the MotifFinder program. Despite its substantially larger size, all summary statistics for the randomized data were consistent with both the experimental and negative results. As examples, skewness (0.31) and kurtosis (-1.8) were effectively identical to the empirical datasets. In addition, the overall match frequencies again exhibited a strong fit to a discrete uniform distribution model (Figure 3C) and the proportion of low accuracy matches (1-4) was consistent (96.5%). Overall, our randomization approach appeared to have effectively modeled the data signal exhibited by the experimental dataset proteins (Figure 2). A total of 34,567 Lysine residues were found in the randomized dataset of which 34,955 were potential motif sites (away from edges). Within the potential sites, 32,795 positive hits (1-7) were recorded. Interestingly, despite the large number of potential sites only one exact match was found to occur by chance within the 999 “proteins” (Figure 2B). An additional 126 high probability sites (6 amino acid matches) were also identified.

Using the rate of occurrence of perfect matches (7 variable amino acids) in the randomized dataset as a crude estimator, we made a first approximation of the probability of encountering 8 motifs within 155 proteins. Based an initial expectation of 1 match per 999 proteins, the joint probability of finding 8 perfect motifs at random within 155 proteins was estimated to be extremely small at ~ 4.0 x 10^-7^ . A similar pattern was found for the near-perfect matches (6 variable amino acids) in the experimental dataset where 26 matches were observed compared to 19.8 predicted. As mentioned, it is obvious that bias did exist within the experimental dataset relate to protein selection, however even if the probability estimates are inflated by 1 or more orders of magnitude the net conclusion remains; the total numbers of perfect and near-perfect motif matches observed were well beyond random expectations.

Collectively, the results provided by comparisons of the experimental, negative and randomized datasets provided evidence in support of our hypothesis that a Lysine-centered ubiquitination motif might exist among TRAF6 / p62 interactors. The overall similarity between the negative and experimental match distributions argues that the selection of potential interacting proteins for the experimental group did not unduly bias the overall distribution pattern. Conversely, the presence of probabilistically unlikely large numbers of perfect and near perfect motif matches within the related experimental group proteins suggested that the motif is by itself or in association with an amino acid sequence of conserved biological importance.

### Analysis of putative ubiquitination site selection

**Figure 4:**
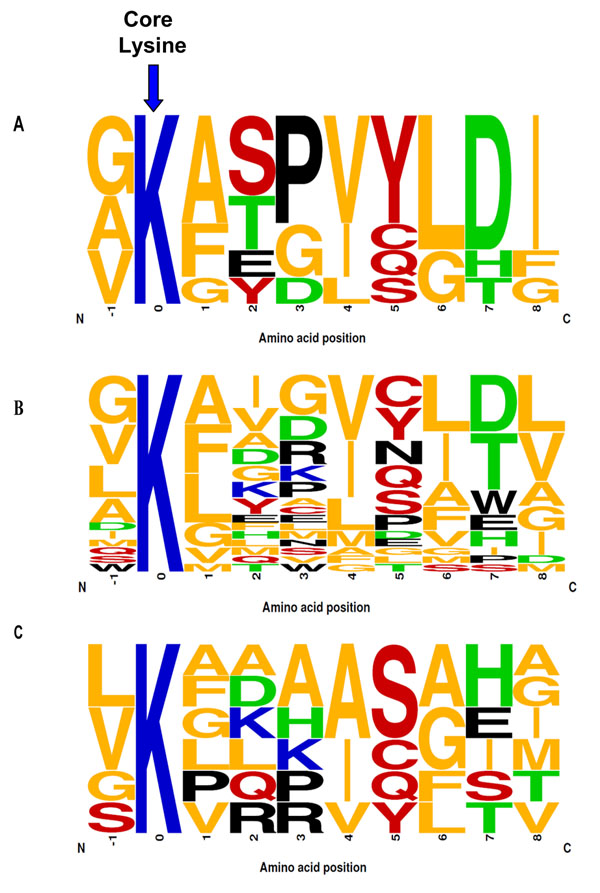
**Sequence logos illustrating the occurrence of amino acids at each site within the putative motif. A:** Frequency distribution of amino acids surrounding Lysines (K) with positive hits at seven variable positions in a ten amino acid long consensus motif in the experimental dataset. **B:** The distribution at six variable positions in a ten amino acid long consensus motif in the experimental dataset. **C:** The distribution at six variable positions in a ten amino acid long consensus motif in the negative dataset. K (red); AFGILMV (blue); CQSY (green); DHT (orange).

Next, we sought to identify amino acids that might play a critical role in ubiquitination site selection and investigated whether there were preferences for certain amino acids near the target ubiquitinated Lysines. This analysis focused on the well-defined proteins from Group I of the experimental dataset. Notably, when we examined the surrounding residues of the validated ubiquitinated Lysine with amino acids conserved at 7 variable positions in our hypothesized motif (perfect hit), we observed an enrichment of small residues (G/A) on the either side of the target site and high frequency of Valine at position 4, Leucine at position 6, and Aspartic Acid at position 7 (Figure 4A). A closer look at all proteins from the experimental dataset (Groups I through V) with amino acids conserved at 6 positions revealed a similar distribution of amino acids, (Figure 4B). When the distribution of positive matches at 6 positions in the negative dataset was evaluated, the pattern of specific amino acids surrounding the core Lysine residue was less consistent (Figure 4C). However, in all datasets, the target Lysine residue was predominantly surrounded by hydrophobic residues (Glycine / Alanine / Valine / Leucine / Isoleucine).

### Secondary structure prediction

Because post-translational modifications tend to be concentrated within specific structural regions of a protein, we further investigated structural constraints of the predicted Lysines. Only predicted Lysines from highly positive (conserved at 6 or 7 variable sites) motif sites were included in this analysis. These Lysines were classified as a high probability group. There was a total of 30 proteins in this category, 25 from the experimental dataset and 5 from the negative dataset. Eight of those 30 proteins had more than one predicted TRAF6/p62 ubiquitination site, such as one 7 match plus one 6 match motif. NRIF (K19), TrkA (K485), TrkB (K811), TrkC (K602 and K815), NTRK2 (K828), NTRK3 (K829) and MBP (K169) were found to possess a perfect match for the amino acid consensus motif for TRAF6/p62 ubiquitination (Table 1). Interestingly of the many Lysines in each of these proteins, only a select Lysine was predicted and/or verified to be ubiquitinated [3,4]. GO ontology analysis of these high probability proteins with perfect match reveled that they were involved mainly in membrane bound signaling events (Table 2).

**Table 1:** Lysines identified as TRAF6 ubiquitination sites and their structural characteristics.

Protein Name	Target Lysine	Secondary Structure	Solvent Accessibility	Disorder Region	Domains Predicted
**TrkA**	485	Loop	Exposed	470 - 490	None
**TrkB**	811	Loop	Exposed	810 – 820	None
TrkC*	602	B strand	Buried	0	Kinase_Tyr
	815	Loop	Exposed	813 – 817	None
**NTRK2**	828	Loop	Exposed	827 – 834	None
**NTRK3**	829	Loop	Exposed	827 – 833	None
**NRIF**	19	Loop	Exposed	13 - 40	KRAB
**MBP**	169	Loop	Exposed	162 – 171	Myelin_MBP

* TrkC contained 2 perfect match motif sites

**Table 2:** GO ontology analysis of sites in proteins with perfect match to the hypothesized motif for TRAF6/p62 ubiquitination.

Protein name	GO: processes	GO: Term for function	GO: function	GO: compotent
TrKA	small GTPase mediated signal transduction, transmembrane receptor protein tyrosine kinase signaling pathway, nervous system development	GO:0005515	protein binding	Plasma membrane, cytosol, endosome
TrkB	transmembrane receptor protein tyrosine kinase signaling pathway, regulation of dendrite development	GO:0005515	protein binding	Plasma membrane, cytosol, endosome
TrkC	transmembrane receptor protein tyrosine kinase signaling pathway, nervous system development	GO:0005515	protein binding	Plasma membrane, cytosol, endosome
NTRK2	nervous system development, transmembrane receptor protein tyrosine kinase signaling pathway, activation of adenylate cyclase activity	GO:0043121, GO:0005515	neurotrophin binding protein binding	Integral to plasma membrane, cytoplasm
NTRK3	nervous system	GO:0043121,	neurotrophin binding	Integral to plasma
	development, transmembrane receptor protein tyrosine kinase signaling pathway, activation of adenylate cyclase activity	GO:0005515	protein binding	membrane, cytoplasm
NRIF	regulation of transcription	GO:0005520	protein binding	Nucleus
MBP	synaptic transmission, central nervous system development, central nervous system development	GO:0019911	structural constituent of myelin sheath	Plasma membrane

**Figure 5:**
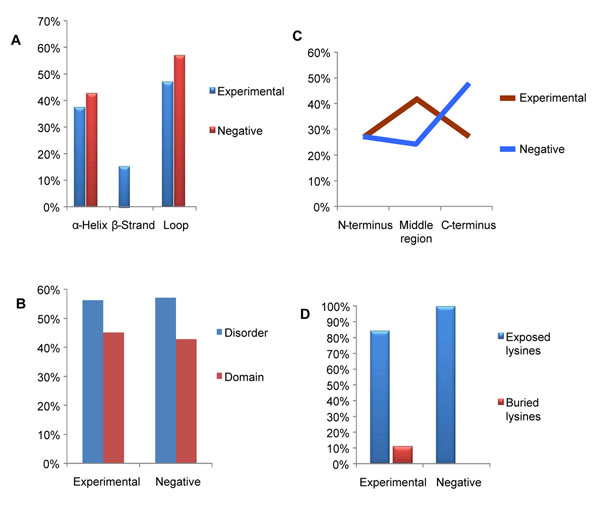
**Structural context of predicted TRAF6/p62 ubiquitination sites within 30 proteins containing perfect motif matches. A:** Distribution of perfect match motif sites based on secondary structure for the experimental and negative datasets. **B:** Percentage distribution of predicted sites in disordered protein regions and domain structures. **C:** Relative proportions of perfect match sites found in three protein regions, N-terminus, Middle and C-terminus. **D:** Identification of perfect match Lysines as likely occupying exposed or buried protein regions as determined by solvent accessibility predictions.

We further sought to incorporate sequence information as well as information from sequence derived structural features of these proteins into the validation process. To do so, four potential structural features of the predicted high probability sites were evaluated: secondary structure, relative distribution within the protein, solvent accessibility, and the intrinsic disorder within the protein domain. Structural analysis was conducted using secondary structure, protein domain, and disorder prediction algorithms [25]. Our results indicated that approximately one-half of the ubiquitination sites were predicted to be in loops (Figure 5A) and disordered regions (Figure 5B). Beta-sheets had the least representation of predicted ubiquitination sites (with 15% sites in experimental and none from negative datasets). The predicted ubiquitinated site was found at a significantly greater rate in the loop regions than in the beta sheets of the protein structure (*P* = 0.0001). The second most common secondary structure was an alpha-helix (Figure 5A). Alpha helices and loops are usually found on the surface of proteins and are tend to easily accessible for post-translational modifications. The predicted sites showed significantly high occurrence of sites in helices and loops as compared to beta sheets (*P* = 0.0001). This was in agreement with previously reported findings on preferred *in vivo* ubiquitination sites in yeast proteins [28]. As an example where surface accessibility has been shown to define a ubiquitination site, position of Lysine 507 of Smad4 is ubiquitinated in the fully solvent-accessible L3 loop with its side chain protruding from the L3 loop surface to the neighboring space [29].

### C-terminal Lysines

The highest possible resolution for investigating structure–function relationships is that of individual residues and their corresponding microenvironments [30]. To provide information on this aspect of hypothetical high-probability sites, the distribution of predicted Lysines residues with regards to their relative position within the protein sequence was evaluated (Figure 5C). Nearly half (48%) of the core motif Lysines were located near the C-termini of the proteins in the experimental dataset as compared to only 28% in the negative dataset. The remaining predicted sites were evenly distributed (25.8%) at the C-terminus or middle region of the proteins in the experimental dataset. On the contrary, within the negative dataset most (42%) target Lysines were located in the middle region of the protein (Figure 5C). This could be either because of false positive prediction of the sites or due to true positive (valid) sites that are buried inside the protein and become exposed when these proteins undergo conformational changes induced by other post-translational modifications or protein-protein interactions. This finding was consistent with studies of the TRAF6 substrate, IRF7 that is ubiquitinated at multiple sites both *in vitro* and *in vivo* with the three C-terminal Lysines (positions 444, 446, and 452) essential for activation of IRF7 [31,32]. Similar studies on SUMOylation sites of LEDGF/p75 have shown that K75, K250, and K254 mapped on the N-terminal region located in evolutionarily conserved charge-rich regions, while C-terminal K364 was identified as solvent exposed [33]. There were 98 Lysines in the N-terminal regions of the proteins in the experimental dataset that were not recognized by the program as they lacked the required 8 amino acids towards the N-terminal end to fit the 10 amino acid long motif. Out of these 98 Lysines, there were five instances of di-Lysines and four tri-Lysines with one occurrence of a poly-Lysine chain of 9 Lysines. The negative dataset on the other hand had 11 N-terminal Lysines, with only one occurrence of a di-Lysine. No specific amino acid distribution pattern was observed surrounding the N-terminal Lysines. The downstream Lysines in the di-Lysine sequences have been reported to be preferentially ubiquitinated [28].

### Surface accessibility

Recent studies of all post-translationally modified proteins documented in Swiss-Prot have shown that most reversible modifications are found on protein surfaces [34]. Ubiquitinated Lysines are surface exposed but this information is hidden in the primary sequence of the protein which can be detected by a surface accessibility predictor. To examine this possibility for the data, solvent accessibility of the high probability core Lysines for modification was examined. Solvent accessibility of an individual residue is often classified as “buried" or "exposed" using geometric analysis (geometric similarity in the arrangement of the water molecules around proteins) [35] or predictive methods. Prediction of solvent accessibilities revealed 84% of the highly positive sites in the experimental dataset and 100% of the negative dataset were exposed on the surface of the protein, which in a cellular environment, would be easily accessible to the active TRAF6/p62 complex (Figure 5D). It has been reported that surface accessibility of post-translational modifications is important for protein-protein interactivity [34].

### Compartment specific ubiquitination motif

**Figure 6:**
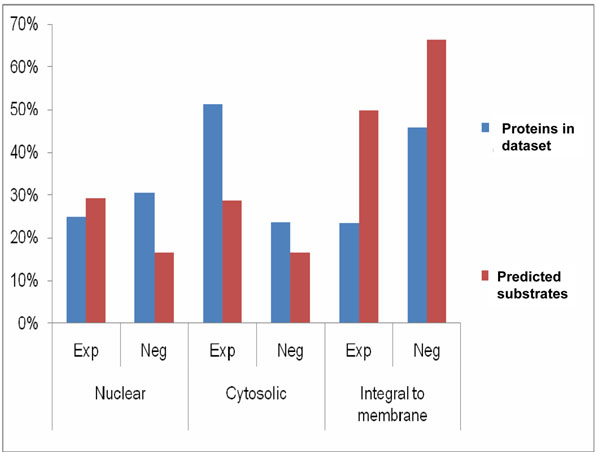
**Sub-cellular localization of TRAF6/p62 substrates.** Comparisons presented for each database showing the total proportion of proteins in each category versus the proportion predicted among the perfect and near perfect motif match proteins.

To study the subcellular distribution of the predicted TRAF6/p62 substrates, compartmentalization of the proteins in both the datasets was examined (Table 2, Figure 6). Proteins were assigned to cellular compartments based on the literature, curated information in protein databases and GO ontology for protein subcellular localization [36].

Localization data of the high probability substrates revealed that relatively few cytosolic proteins were predicted to be TRAF6/p62 substrates. However, when the nuclear proteins in the experimental dataset were examined, the proportion predicted to function as TRAF6/p62 substrates (29%) was slightly higher than the total percentage of nuclear proteins (25%) found in the dataset (Figure 6). A substantial increase in prediction percentages was, however, observed for proteins that were integral to membranes in both the experimental and negative datasets. This finding suggests that either our motif was biased in favor of a pattern that occurs more commonly within membrane bound proteins, or potential that a meaningful cellular association was elucidated. Since the consensus motif was based on plasma membrane bound TrkA and nuclear protein NRIF [3,4], we hypothesize that a combination of both conclusions may be correct. Further refinement of the motif as more substrates are experimentally verified from various subcellular localizations will be needed to clarify this observation.

### Sequence conservation

We sought to further validate the biological relevance of our hypothetical ubiquitination motif by examining it in an evolutionary context. For this analysis a high-confidence set of TRAF6/P62 substrates, those with perfect matches to our putative motif, were selected for alignment. These proteins were TrkA, TrkB, TrkC, NRIF, NTRK2, NTRK3 and MBP (Table 1). To check for potential evidence of evolutionary pressure to conserve the site-specific ubiquitinated Lysines, conservation of our predicted sites in these eight proteins was examined across multiple species [15]. Our results indicated that a predicted ubiquitination sites were conserved from among six mammalian species (*data not shown*). This unusually high conservation suggests that the ubiquitination of these sites may potentially be conserved across a wide spectrum of life forms, although this remains untested. A high degree of conservation among proteins that are ubiquitinated also suggests that they may have arisen early in the course of evolution. However, a significant number of ubiquitination sites differ in the ubiquitome and the extent of homology is not uniform because of the high diversity among the proteins. Nevertheless, evidence of conservation does suggest that ubiquitination is in each case indispensable for protein function, which is in turn essential for regulating cellular function. These highly conserved essential ubiquitination events may reflect how early forms of life used protein ubiquitination in specific housekeeping cellular functions. Interestingly, our results indicated that although the surrounding sequence regions may diverge, the critical residues remain conserved. Similar whole genome-scale studies have shown that 2,683 potential SUMO substrates are conserved between human and mouse based on pattern recognition and phylogenetic conservation [37]. In another study, linear pattern recognition in combination with phylogenetic conservation was first used to discover transcription factor binding sites [38]. This report was similar to recent studies on phosphorylation sites which demonstrated similar conservation within protein families [39], thus pointing at generic regulatory mechanisms which may be conserved across species. The presence of evolutionary conservation is mechanistically important because the short lengths and rarity within a complex proteome make linear motifs difficult to find computationally [40].

## Discussion

Most proteins in cells undergo post-translational modifications giving them structural and functional diversity for diverse roles in biological processes. Experimental identification and validation of posttranslational modifications (PTMs) is labor-intensive task and can be expensive in the absence of prior knowledge concerning PTMs. Analyzing the ‘ubiquitome’ is one of the most exciting and challenging tasks in current proteomics research. A lack of curated datasets of ubiquitinated proteins presents the ultimate limiting factor in studying substrate selection mechanism in ubiquitination making it difficult to evaluate, and compare target sites. As more and more ligases are identified there exists an urgent need to rapidly and precisely identify enzyme-specific substrates to decode their selectivity and specificity [8]. Computational prediction of PTM sites has provided researchers with information on the high probability PTM sites for further experimental characterizations like PHOSIDA and NetPhos for phosphorylation [41,42], SUMOsp for SUMOylation [43] and NetAcet for prediction of N-acetyltransferase A substrates [44].

In this study, a computational tool was developed to predict Lysine ubiquitination sites from sequences using MATLAB programs and online prediction software. As more validated ubiquitinated sites from experimental data become available, and appropriate changes are made based on the available site data, further predictions can be made. The inclusion of structural information to improve the prediction tools could be another way to enhance the prediction performance as ubiquitination is an enzymatic process, and the interactions between target sites and enzymes concerned should be structurally satisfied. The model that we propose herein could be applied to other E3 Ub ligases that are known to employ scaffold proteins to aid in their substrate selection process. One such example is DYRK2–EDVP E3 ligase complex where DYRK2 not only is it serves as adaptor for assembly of the active Ub ligase complex, but it also phosphorylates its substrate and primes the substrate for degradation [45]. Thus, use of bioinformatics methods to predict site modification *in silico* could yield more defined results*.* These prediction tools should be closely integrated into the interpretation of proteomic experiments.

Here we identified the interactome of the active enzyme complex and studied the verified substrates for characterization of target sites to predict substrates. As proteomics methods identify additional *in vivo* ubiquitination sites, prediction algorithms can be fine tuned and improved. A conserved motif that serves as a recognition determinant for TRAF6/p62 enzyme complex was identified. Studies have shown some structural preferences for ubiquitination of targeted proteins such as preferred choice of Lysines in loops and/or for easily accessible Lysines within α–helical regions [46]. Our findings indicate a bias towards a specific consensus sequence motif for ubiquitination by TRAF6/p62. Moreover, it appears that the active complex targets an accessible surface residue providing the selection process with a conformational recognition mechanism. We propose that the scaffold, p62, plays an important role in recruiting substrates for TRAF6, thereby facilitating interaction with an accessible Lysine residue in a loop or helical structures on the surface of the substrate resulting in K63-polyubiquitination at a specific Lysine, if the flanking residues fit the consensus motif. In this regard, it is of interest to point out that the scaffold TAB2 and TAB3, although ubiquitinated by TRAF6 (7), did not possess the motif identified by our search algorithm. This finding supports the notion that specific interaction of the ligase with a given scaffold may facilitate substrate ubiquitination in response to a given external stimulus.

A total of 42 high probability TRAF6/p62 ubiquitination sites in 30 proteins were identified by our prediction approach. Structural analysis of these predicted TRAF6/P62 substrates showed that the predicted ubiquitination sites were biased towards the C-terminal domain of the protein [31,32]. Secondary structure analysis of the predicted sites revealed overall preference for loops and helices than beta-strands and solvent accessibility analysis of predicted Lysines revealed most of the predicted sites were exposed on the surface of the protein rather than being buried. There was high structural and phylogenetic conservation of predicted sites. Tertiary structure analyses of investigated proteins revealed that most of the predicted sites are likely to be exposed on the surface of the protein rather than being buried. Although linear conservation of individual amino acids within the consensus motif at the predicted ubiquitinated sites is low, there is a high structural and evolutionary conservation of predicted sites across mammalian species. The high accessibility of ubiquitination sites suggests that they are localized in loops and helices, since these structural elements are usually found at the protein surface. It is well known that the loop regions frequently participate in forming binding sites and active sites of enzymes making them excellent substrates for regulation [40]. Beta sheets can be internal to a protein (largely hydrophobic) or on the surface in which case they are amphipathic, with every other amino acid side chain alternating between hydrophobic and hydrophilic nature. Because post-translational modification sites are predominantly located in rapidly evolving loop regions [40], relaxed evolutionary constraints on loops allow them to evolve rapidly and rather independently from the protein core [41]. Formally, disordered regions are defined as regions within proteins that lack a precise 3D structure and consist of an ensemble of fluctuating, interconverting conformers. These regions have been known to be associated frequently with posttranslational modifications [48]. Disorder prediction of linear motifs and their flanking regions for the experimentally characterized examples from the Eukaryotic Linear Motif (ELM) database revealed that short recognitions motifs are embedded in locally unstructured regions [48]. Thus, structurally and evolutionarily, our high-confidence set of TRAF6/62 substrates and highly positive motif sites represent a reasonable site for post-translational modification by ubiquitin.

Findings from this study indicate that the ubiquitination site prediction is closely correlated with the amino acid property around the ubiquitination site. Our approach makes it possible to find putative novel ubiquitination sites that have not (yet) been experimentally identified. Thus, in the absence of experimental data, the prediction of novel ubiquitination sites can be taken as the first method of an experimental design for uncovering functionality of any protein of interest and elucidating its involvement in certain signaling cascades. Methods for computational prediction of peptide specificities and identification of substrates could be enhanced by combining different approaches and integrating various types of information. In addition, the prediction approach taken here combined with experimental verification will propel our understanding of ubiquitination mechanisms. Thus, a combination of both a computational and experimental approach could propel our understanding of ubiquitination dynamics into a new phase.

## Conclusions

In conclusion, NRIF (K19), TrkA (K485), TrkB (K811), TrkC (K602 and K815), NTRK2 (K828), NTRK3 (K829) and MBP (K169) were found to possess a perfect match for the amino acid consensus motif for TRAF6/p62 ubiquitination. The ability of a given Lysine residue to serve as a *bona fide in vivo* ubiquitination site has been verified by mutagenesis along with verification of K63-ubiquitin linked chains for sites observed in TrkA, B, C, and NRIF [3,4]. NTRK2, NTRK3 and MBP are putative TRAF6 substrates which have not been previously reported to interact with p62 and thus define new signaling networks for integration of this E3/scaffold. Altogether these findings suggest that the approach we describe could be applied to other E3 ligases for prediction of their substrates taking into account the specificity provided by the scaffold which aid in the formation of the signalsome in response to external stimuli.

## List of abbreviations

AID: Atypical PKC-interaction domain; aPKC: Atypical protein kinase C; ELM: Eukaryotic linear motifs; FA: Formic acid; K: Lysine; KO: Knock-out; MBP: Myelin basic protein; NEMO: NF-kappa-B essential modulator; NRIF: Neurotrophin receptor interacting factor; NTRK2: Neurotrophic tyrosine receptor kinase 2; NTRK3: Neurotrophic tyrosine receptor kinase 3; PB1: Phox and Bem1; PEST: Proline Glutamate Serine Threonine; PTM: Post translational modification; RING: Really Interesting New Gene; SUMO: Small ubiquitin-related modifier; TAB2: TGF-beta activated kinase 1/MAP3K7 binding protein 2; TAB3: TGF-beta activated kinase 1/MAP3K7 binding protein 3; TRAF6: Tumor necrosis factor receptor associated factor 6; TrkA: Tropomyosin receptor kinase A; TrkB: Tropomyosin receptor kinase B; TrkC: Tropomyosin receptor kinase C; Ub: Ubiquitin; UBA: Ubiquitin-associated domain; ZIP: Zeta protein kinase C interacting protein; ZZ: ZZ-type Zinc finger domain

## Competing interests

The authors declare that they have no competing interests.

## Authors’ contributions

TSJ identified the consensus motif, assembled the protein datasets, compiled the search results, conducted the secondary structure analyses, conducted the SLiMSearch analysis, generated the randomized protein dataset, conducted parts of the statistical analyses and drafted the manuscript. MCW developed and programmed the MotifMaker and MotifFinder algorithms, conducted the motif searches using these programs, developed the probability estimates and participated in drafting of the manuscript. MWW conceived the study, led the project design, provided technical direction for the research and participated in drafting of the manuscript. All authors read and approved the final manuscript.
